# Call for Organization of a District Society

**Published:** 1894-07

**Authors:** 


### Society Notes.

### Call for Organization of a District Society.

Bowie, Texas, June 30, 1894.

Dear Doctor:—Realizing the very great necessity of a medical association, conveniently located to the physicians along the ines of the Fort Worth & Denver and the Rock Island Railroads, and adjacent country between Fort Worth and Amarillo on the Denver, and Duncan on the Rock Island, we have, after very careful consideration, decided to invite the physicians in said territory to meet at the office of Dr. C. C. Davis, in Bowie, Texas, July 17th, at 10 o'clock a. m., to perfect such organization, and consider the propriety of permanently locating it in Bowie, this place being centrally located, and very accessible.

We very earnestly solicit you to meet with and give us your aid in this all-important matter.

Knowing the character of the material that will compose this Association, we feel that it cannot but be a grand success. Please notify Dr. C. C. Davis, Bowie, Texas, as early as possible, if you will attend on the above date.

Trusting this move will meet your approval and have your hearty support, we are,

Yours fraternally,

W. L. York,
C. S. Bobo,
W. A. Morton,
T. R. Allen,
J. T. Sparkman,
B. C. Mitchell,
C. C. Davis.

